# Endothelial angiopoietin-2 overexpression in explanted livers identifies subjects at higher risk of recurrence of hepatocellular carcinoma after liver transplantation

**DOI:** 10.3389/fonc.2022.960808

**Published:** 2022-09-08

**Authors:** Simone Lasagni, Filippo Leonardi, Alessandra Pivetti, Lorenza Di Marco, Federico Ravaioli, Matteo Serenari, Stefano Gitto, Rosina Maria Critelli, Fabiola Milosa, Adriana Romanzi, Serena Mancarella, Francesco Dituri, Mattia Riefolo, Barbara Catellani, Paolo Magistri, Dante Romagnoli, Ciro Celsa, Marco Enea, Nicola de Maria, Filippo Schepis, Antonio Colecchia, Calogero Cammà, Matteo Cescon, Antonietta d’Errico, Fabrizio di Benedetto, Gianluigi Giannelli, Maria Luz Martinez-Chantar, Erica Villa

**Affiliations:** ^1^ Gastroenterology Unit, Department of Medical Specialties, University of Modena and Reggio Emilia and Azienda Ospedaliero-Universitaria di Modena, Modena, Italy; ^2^ Clinical and Experimental Medicine PhD Program, University of Modena and Reggio Emilia, Modena, Italy; ^3^ Gastroenterology and Transplant Hepatology, Papa Giovanni XXIII Hospital, Bergamo, Italy; ^4^ Liver Transplant Center, University of Bologna, Balogna, Italy; ^5^ Department of Experimental and Clinical Medicine, University of Florence, Florence, Italy; ^6^ National Institute of Gastroenterology “Saverio de Bellis”, Research Hospital, Castellana Grotte, Italy; ^7^ Pathology Unit, Istituto di ricovero e cura a carattere scientifico (IRCCS) Azienda Ospedaliero-Universitaria di Bologna, Bologna, Italy; ^8^ Liver Transplant Center, University of Modena and Reggio Emilia and Azienda Ospedaliero-Universitaria di Modena, Modena, Italy; ^9^ Section of Gastroenterology and Hepatology, Department of Health Promotion, Mother and Child Care, Internal Medicine and Medical Specialties (PROMISE), University of Palermo, Palermo, Italy; ^10^ Department of Surgical, Oncological and Oral Sciences (Di.Chir.On.S.), University of Palermo, Palermo, Italy; ^11^ Internal Medicine and Medical Specialties, Department of Health Promotion, Mother and Child Care (PROMISE) University of Palermo, Palermo, Spain; ^12^ Liver Disease Laboratory, Center for Cooperative Research in Biosciences (CIC bioGUNE), Basque Research and Technology Alliance (BRTA), Derio, Spain; ^13^ Centro de Investigacion Biomedica en Red de Enfermedades Hepaticas y Digestivas (CIBERehd “Instituto de Salud Carlos III”), Derio, Spain

**Keywords:** hepatocellular carcinoma, liver transplantation, recurrence, angiopoietin-2, survival, neoangiogenesis, immunocytochemistry

## Abstract

**Background:**

Though the precise criteria for accessing LT are consistently being applied, HCC recurrence (HCC-R_LT) still affects more than 15% of the patients. We analyzed the clinical, histopathological, and biological features of patients with HCC to identify the predictive factors associated with cancer recurrence and survival after LT.

**Methods:**

We retrospectively analyzed 441 patients with HCC who underwent LT in our center. Overall, 70 (15.8%) of them developed HCC-R_LT. We matched them by age at transplant and etiology with 70 non-recurrent patients. A comparable cohort from the Liver Transplant Centre of Bologna served as validation. The clinical and biochemical characteristics and pre-LT criteria (Milan, Metroticket, Metroticket_AFP, and AFP model) were evaluated. Histological analysis and immunohistochemistry for angiopoietin-2 in the tumor and non-tumor tissue of explanted livers were performed. Patients’ follow-up was until death, last clinical evaluation, or 31 December 2021. In patients with HCC-R_LT, the date of diagnosis of recurrence and anatomical site has been reported; if a biopsy of recurrence was available, histologic and immunohistochemical analyses were also performed.

**Results:**

Patients were followed up for a mean period of 62.7 ± 54.7 months (median, 39 months). A higher risk of HCC-R_LT was evident for factors related indirectly (AFP) or directly (endothelial angiopoietin-2, microvascular invasion) to biological HCC aggressiveness. In multivariate analysis, only angiopoietin-2 expression was independently associated with recurrence. Extremely high levels of endothelial angiopoietin-2 expression were also found in hepatic recurrence and all different metastatic locations. In univariate analysis, MELD, Metroticket_AFP Score, Edmondson–Steiner grade, microvascular invasion, and endothelial angiopoietin-2 were significantly related to survival. In multivariate analysis, angiopoietin-2 expression, Metroticket_AFP score, and MELD (in both training and validation cohorts) independently predicted mortality. In time-dependent area under receiver operating characteristic curve analysis, the endothelial angiopoietin-2 expression had the highest specificity and sensitivity for recurrence (AUC 0.922, 95% CI 0.876–0.962, *p* < 0.0001).

**Conclusions:**

Endothelial angiopoietin-2 expression is a powerful independent predictor of post-LT tumor recurrence and mortality, highlighting the fundamental role of tumor biology in defining the patients’ prognosis after liver transplantation. The great advantage of endothelial angiopoietin-2 is that it is evaluable in HCC biopsy before LT and could drive a patient’s priority on the waiting list.

## Introduction

Liver cancer remains a major global health problem, representing a leading cause of cancer deaths worldwide (1). Hepatocellular carcinoma (HCC) is the most common type of primary liver cancer (75%). The incidence of HCC increases progressively with age in all populations, with a peak at 70 years, and the male-to-female ratio is estimated to be 2–2.5:1 (2). Approximately 90% of HCC cases occur in the setting of known underlying etiology, most frequently chronic viral hepatitis (HBV and HCV), alcohol intake, and dysmetabolism; in the Western world, the most considerable attributable fraction is caused by chronic hepatitis C, although data are changing with the advent of the new direct-acting antiviral drugs for HCV. The presence of cirrhosis is associated with a significant risk of HCC, with approximately one-third of the cirrhotic patients developing HCC during their lifetime, and liver diseases’ severity correlates with risk for HCC development (3, 4). One of the scientific community’s main objectives is, therefore, to identify the best therapeutic option for HCC patients, in the complexity of a patient affected not only by a neoplasm but also by liver disease, which is sometimes profoundly disabling.

Liver transplantation (LT) is currently the only effective potential cure for patients with HCC and underlying liver disease. The criteria for access to LT for HCC patients have been continuously refined in the attempt to reduce the HCC recurrence rate and, thus, increase post-LT survival. In 1996, Mazzaferro and colleagues demonstrated that HCC patients with well-defined characteristics (Milan criteria: single tumor ≤ 5 cm or maximum of three nodules ≤ 3 cm in the absence of vascular invasion) had a much better survival rate after LT than patients who exceeded these limits, comparable to patients transplanted for non-neoplastic conditions (5-year survival of 75%) (5). The Milan criteria were then acquired in the “Barcelona Cancer Liver Classification (BCLC)”, universally adopted as a gold standard in HCC therapy, as guidelines for access to liver transplantation (6).

Currently, the presence of HCC is the fastest-growing indication of LT worldwide (7). In recent years, many authors have therefore tried to find new LT criteria for HCC that extended the Milan criteria, in order to make available a curative treatment to a greater number of patients, with comparable survival and tumor recurrence rates. To achieve this goal, “Downstaging” loco-regional treatments for HCC outside the Milan criteria and the search for “Extended Criteria” were studied.

Among the best known, also externally validated, were the San Francisco criteria (single nodule ≤6.5 cm or two to three nodules ≤4.5 cm with a total diameter ≤8 cm) (8), the Up-To-Seven criteria [HCC which has 7 as the sum of the diameter (cm) of the major nodule and the number of nodules] (9), and the French-AFP model (point system based on tumor diameter, number of nodules, and AFP value) (10). The 5-year survival rates estimated using these criteria were 75.2% (8), 71.2% (9), and 69.9% (10), respectively. For all these scores, the percentages of neoplastic recurrence (approximately 15%–20% of the total cases) were very similar to the standard criteria. Recently, Mazzaferro and colleagues developed a new model based on the AFP level, size, and number of nodules able to estimate at any time (pre- or post-downstaging) the probability of 5-year post-LT HCC recurrence-free survival—the Metroticket 2.0 Model (11).

Despite all these refinements, about 15%–20% of patients undergoing LT for HCC still suffer from recurrence, which dramatically affects the prognosis (12). This residual percentage of recurrences evades the usual diagnostic systems, not being wholly perceived by the predictive scores mentioned above or by other risk factors such as the biological characteristics of the tumor, i.e., the Edmondson–Steiner grade or the presence of microvascular invasion (13).

This substantial residual rate of recurrences, regardless of the score used to predict risk, suggests that other factors besides the number and size of lesions should be considered when assessing the risk of recurrence. The biology of the tumor is undoubtedly one of these. Still, despite solid evidence of the role of HCC aggressiveness in determining its clinical course (14, 15), this has not been evaluated in the transplant setting. The aggressiveness of HCC can be defined in several ways, the grading system of Edmondson–Steiner (E-D score) being the most feasible and available for all transplant centers (16, 17). However, in addition to not being included in any of the scores above mentioned, the accuracy of the E-D score in identifying aggressive cases is not complete, as a substantial proportion of the E-D score 1–2 is already biologically aggressive when this feature is evaluated by other approaches, such as neoangiogenic transcriptomic signature (14). This signature was set up on fresh tumor tissue, which is often not available in retrospective series, but we have previously shown that the neoangiogenic transcriptomic signature is equivalent to the immunohistochemical expression of angiopoietin-2 (ANGPT2) in HCC tissue (15).

The current study analyzed the clinical, histopathological, and biological features of patients with HCC undergoing liver transplantation at our center in order to identify the role of the biological aggressiveness of tumors in post-transplant HCC recurrence and survival.

## Methods

### Patients

We retrospectively analyzed 441 consecutive HCC patients who underwent LT for HCC between 2000 and 2020 at Modena Liver Transplant Centre (Italy). On the whole, 70 patients (15.8%) experienced recurrence. They were matched by age at LT and etiology with 70 other non-recurrent patients and considered a derivation cohort. As a validation cohort, we identified a cohort of 60 patients who had received liver transplants at the Bologna Liver Transplant Center (Italy) at the same time interval with an HCC recurrence:non-recurrence ratio of 1:1 and matched by age at LT and etiology. We collected data before LT (gender, etiology of liver disease, Child–Pugh and MELD scores, AFP levels, number of HCC lesions and size of the largest lesion, and downstaging treatments for HCC) and at LT (age, number of HCC lesions and size of largest lesions, AFP levels, microvascular invasion, and Edmondson–Steiner grading in the explanted liver and immunosuppressive regimens after LT). The Milan criteria, AFP model (10), and Metroticket_AFP were assessed according to the radiological and biochemical features before LT, while Metroticket (9) was calculated according to the pathology features at LT. The maintenance immunosuppressive schedules were also recorded.

### Outcomes

Outcomes were HCC recurrence and overall survival (OS) after LT. Diagnosis of HCC recurrence was based on a dynamic CT or MRI according to the European Association for the Study of Liver (EASL) guidelines (2). All patients were followed up after LT with clinical examination, biochemical evaluation, and abdominal ultrasound every 3 months and dynamic computed tomography (CT) or magnetic resonance imaging (MRI) every 3 months for the first 3 years after LT.

Patients who developed HCC recurrence were defined as the recurrent group, and those who did not develop HCC recurrence were defined as the non-recurrent group. The patients’ follow-up was until death, last clinical evaluation, or 31 December 2021.

### Immunohistochemical methods

The expression of ANGPT2 in HCC tissue and the surrounding non-tumoral cirrhotic tissue of matched patients of both training and validation sets was evaluated both in hepatocytes and in the vascular endothelia in formalin-fixed paraffin-embedded samples from liver tissue explanted at transplant by routine histologic and immunohistochemical analysis, as already detailed in (15). In nine HCC recurrent patients, tumoral liver or extra-hepatic tumoral tissue (mostly lung metastasis) was also studied. After deparaffinization and rehydration, antigen unmasking was performed with 1 mM EDTA buffer pH 8 at 98°C for 15 min. The sections were then incubated in 5% methanol and 1% H_2_O_2_ for 5 min for blocking endogenous peroxidases, and then nonspecific sites were blocked using a blocking solution reagent with 3% BSA for 30 min at room temperature. Sections were then incubated with primary antibody Goat anti-ANGPT2 (AF623) (R&D Systems, Inc., Minneapolis, MN, USA) at a working dilution of 1:50. Next, the sections were incubated with secondary antibody OmniMap anti-Goat HRP conjugated prediluted (Ventana Medical Systems, Inc., Tucson, AZ, USA), for 20 min in a humidity chamber and then with detection kit reagents [Ultra view universal HRP multimer and diaminobenzidine (DAB) Chromogen, Ventana Medical Systems, Inc., Tucson, AZ, USA] following the manufacturer’s instructions. After this, the sections were counterstained with hematoxylin, dehydrated, and permanently mounted for microscopic examination. Images of the stained liver tissue were processed with ImageJ software (http://rsbweb.nih.gov/) to obtain the medium intensity value of the DAB signal.

### Statistical analysis

Continuous variables were expressed as means ± SD, and data were reported as counts and percentages. Continuous and categorical variables were compared using Student’s *t*-test and the Pearson chi-squared test, respectively.

The Kaplan–Meier method was used to estimate the cumulative probability of HCC recurrence and OS. A log-rank test assessed the differences in observed probability.

Univariate and multivariate Cox regression analyses were used to identify variables associated with HCC recurrence and mortality after LT in matched patients. Robust standard errors were obtained to consider correlation potentially induced by matched observations. The following variables, all obtained at the time of transplant, were evaluated in the univariate analysis: gender, BMI, log AFP at transplant, number of downstaging treatments, Edmondson–Steiner grading, Milan criteria (5), Metroticket and Metroticket_AFP (9), AFP model (10), microvascular invasion in the explant, and endothelial angiopoietin-2 in the explant. Variables with a *p*-value <0.10 at univariate analysis were included in the multivariate models. To prevent a high level of interaction between the different variables, these were tested for collinearity, and we excluded the collinear variables from the multivariate model. For recurrence, we tested the two strongest variables (endothelial angiopoietin-2 and microvascular invasion in the explant), which were collinear, in two different multivariate models, one including endothelial angiopoietin-2 (**Table 1**, first model) and the other microvascular invasion in the explanted liver (**Table 1**, second model).

**Table 1:** Univariate and multivariate analysis results for recurrence of HCC after LT in the training cohort.

Variables	Univariate analysis		Multivariate analysis	
	HR (95% CI)	*p*	HR (95% CI)	*p*
Model 1				
Gender	1.659 (0.716–3.842)	0.237		
BMI	0.916 (0.844–0.994)	0.035	0.940 (0.839–1.052)	0.283
MELD score	1.012 (0.972–1.053)	0.570		
Log AFP LT	1.517 (1.144–2.012)	0.004	0.828 (0.487–1.408)	0.828
Endothelial angiopoietin-2*,**	4.411 (2.352–8.272)	<0.001	5.634 (2.597–12.224)	<0.001
Edmondson–Steiner grade*	2.130 (1.460–3.106)	<0.001		
Microvascular invasion**	3.246 (6.2920–11.904)	<0.001		
Milan Criteria	1.886 (1.167–3.049)	0.010	1.088 (0.505–2.347)	0.829
Metroticket Score	0.998 (0.955–1.042)	0.920		
Metroticket_AFP Score	2.426 (1.387–4.244)	0.002	1.724 (0.779–3.815)	0.179
AFP model	1.279 (1.107–1.477)	<0.001	1.266 (0.963–1.665)	0.092
Number of downstaging treatments before LT	1.091 (0.783–1.520)	0.606		
Model 2				
BMI	0.916 (0.844–0.994)	0.035	0.868 (0.782–0.963)	0.004
Edmondson–Steiner grade	2.130 (1.460–3.106)	<0.001	1.341 (0.804–2.238)	0.261
Microvascular invasion	3.246 (6.2920–11.904)	<0.001	3.676 (1.781–7.588)	<0.001
Milan Criteria	1.886 (1.167–3.049)	0.010	1.154 (0.581–2.292)	0.682
Metroticket_AFP Score	2.426 (1.387–4.244)	0.002	1.360 (0.675–2.743)	0.390
AFP model	1.279 (1.107–1.477)	<0.001	1.090 (0.845–1.405)	0.508

*, **, collinear.

The estimated survival function was computed to assess the expected time to HCC recurrence and survival after LT for a hypothetical patient with a combination of prognostic factors. Time-dependent area under receiver operating characteristic curve (AUROC) was used to assess the discrimination of the obtained multivariate model and AFP model for HCC recurrence.

PASW Statistics (ver. 28; IBM Corporation, Armonk, NY, USA) was used for the statistical analyses. The study protocol was approved by the Area Vasta Emilia Nord (AVEN) Ethics Committee (IRB10/08_CE_UniRer/AOU_MO).

## Results

Patients were followed up for a mean period of 62.7 ± 54.7 months (median, 39 months). Recurrence occurred after 27.1 ± 34.9 months (median, 15 months) from LT. All recurrent patients but 6 (64, 91.4%) as well as 60 non-recurrent patients (85.7%) were men (*p* = 0.288 Fisher’s exact test). By matching the mean age at LT and the etiology of primary liver disease, it was observed that there were no significant differences between the two groups. The mean AFP values at HCC diagnosis were not significantly different between recurrent and non-recurrent patients (*p* = 0.103, Mann–Whitney *U* test) while the AFP levels were significantly higher at the time of LT in the recurrent group than in the non-recurrent group (*p* = 0.004). There was no significant difference in recurrence rate in patients undergoing one downstaging treatment, two or more, or no treatment before LT.

In the group of recurrent patients, 35 (50.0%) were Milan-in and 35 (50.0%) were Milan-out; among non-recurrent patients, 18 (25.7%) were Milan-out and 52 (74.3%) were Milan in (*p* = 0.003, Pearson Chi-square). The Metroticket values did not discriminate between recurrent and non-recurrent patients (*p* = 0.307), while the Metroticket_AFP values were significantly different between the two groups (*p* < 0.001) (**Table 2**). The distribution of different scores in the AFP model between recurrent and non-recurrent patients was of borderline significance (*p* = 0.049).

**Table 2:** Demographic, clinical, pathological, and histopathological features of training (Liver Transplant Centre of Modena) (*n* = 140) and validation (Liver Transplant Centre of Bologna) (*n* = 60) cohorts at the time of liver transplant for hepatocellular carcinoma (HCC).

Characteristics	Modena’s training cohort (*n* = 140)			Bologna’s validation cohort (*n* = 60)			Intergroup *p*-value
	Recurrent group (*n* = 70)	Non recurrent group (*n* = 70)	Intragroup *p*-value	Recurrent group (*n* = 30)	Non recurrent group (*n* = 30)	Intragroup *p*-value	
Male gender (*n*, %)	64 (91.4)	60 (85.7)	0.288	25 (86.2)	26 (86.7)	0.003	0.484
Age at LT (mean ± SD)	57.2 ± 7.9	57.6 ± 7.4	0.751	59.2 ± 5.4	58.6 ± 5.3	0.868	0.692
Deaths	54 (89.4)	8 (11.4)	<0.0001				
BMI (kg/m^2^) (mean ± SD)	24.6 ± 2.8	26.3 ± 3	0.003	24.9 ± 3.0	26.6 ± 3.3	<0.001	0.148
AFP (ng/ml) (mean ± SD)	377 ± 2631	79.0 ± 94.8	0.011	68.9 ± 125.5	26.7 ± 87.5	0.042	0.107
Tumor endothelial angiopoietin-2	0.43 ± 0.05	0.34 ± 0.05	<0.001	0.44 ± 0.01	0.40 ± 0.01	0.001	0.352
Number of HCC lesions at LT (*n*, %)			1.000			0.217	0.407
≤2	57 (81.4)	57 (81.4)		22 (78.6)	22 (73.3)	
>2	13 (18.6)	13 (18.6)		6 (21.4)	8 (26.7)	
Size of largest lesion at LT (mm) (mean ± SD)	28 ± 16	20 ± 13	<0.001	32.0 ± 23.3	20.2 ± 17.9	0.045	0.088
Microvascular invasion at LT (*n*, %)	23 (34.8)	7 (10.0)	<0.001	18 (66.7)	5 (20.8)	0.004	<0.001
Edmondson–Steiner grade at LT (*n*, %)							
GX G1–G2 G3–G4	9 (12.9)	20 (28.6)	0.018	2 (6.9)	6 (19.4)		
	32 (45.7)	34 (48.6)		8 (27.6)	14 (45.2)	0.031	0.017
	29 (41.4)	16 (22.9)		20 (66.7)	10 (33.3)		
Downstaging treatments before LT (*N*, %)							
None	13 (18.6)	11(15.7)	0.882	1 (3.3)	2 (6.6)	0.673	<0.001
>2	23 (32.9)	25 (35.7)		22 (73.3)	25 (83.3)	
<2	34 (47.6)	34 (48.6)		7 (23.3)	3 (10.0)	
Milan criteria In (*n*, %)	35 (50.0)	52 (74.3)	0.003	14 (46.6)	25 (83.2)	0.001	0.585
Up-To-Seven criteria In (*n*, %)	51 (72.9)	64 (91.4	0.18	24 (80.0)	26 (86.6)		0.147
Metroticket_AFP *	83.6 ± 14.9	92.3 ± 7.9	<0.001	81.2 ± 15.4	92.6 ± 3.9	0.003	0.568
AFP model < 2**	61 (87.1)	65 (92.2)	0.260	21 (68.8)	23 (79.3)	0.376	0.002

Categorical data are expressed as numbers (percentages). Continuous variables are expressed as mean ± standard deviation.

LT, liver transplant. BMI, body mass index. AFP, alfa-fetoprotein. SMD: standardized mean difference.

* For Metroticket_AFP, the mean 5-year survival rate ± standard deviation is reported.

** The cutoff of 2 has been used according to Duvoux et al. (10).

No substantial differences were observed between the two cohorts, but the percentage of patients with AFP model scores lower than 2 among recurrent patients was higher in the derivation cohort. The demographic and clinical characteristics of the two cohorts under study are shown in **Table 2**.

### Histological analysis

Analysis of the Edmondson–Steiner score showed that there was an uneven distribution between recurrent and non-recurrent patients, with a higher percentage of G3–G4 grade among recurrent patients (*p* = 0.018) (**Table 2**).

The microvascular invasion was significantly higher in recurrent than in non-recurrent cases (*p* ≤ 0.001) in both cohorts, with a significantly higher percentage in the validation cohort.

### Immunohistochemical analysis

**Figure 1:**
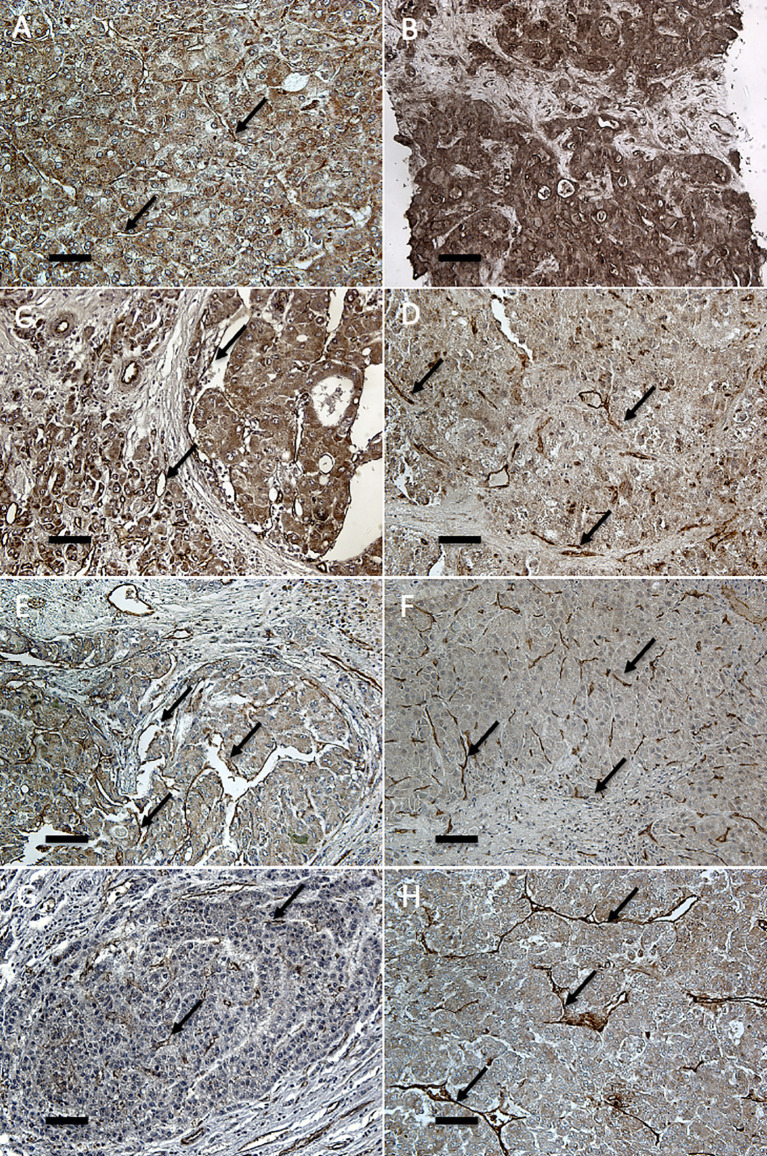
Immunohistochemical analysis of angiopoietin-2 in endothelia of primary and recurrent HCCs after LT. Immunostaining for angiopoietin-2 (20×) shows marked cytoplasmic and vascular endothelial positivity both in primary HCCs at explant **(A, C, E, G)** and in the respective recurrent tumors (**B**: liver; **D**: peritoneum; **F**: lung; **H**: kidney). Endothelial and parenchymal angiopoietin-2 expression in hepatic and extra-hepatic recurrent HCCs is more marked than in the corresponding primary tumor. Arrows indicate representative endothelial localization (scale bar: 6.7 μm).

Immunohistochemical analysis was performed in all explanted livers but four, in which tumoral tissue was completely necrotic and, thus, unsuitable for analysis. The angiopoietin-2 expression in hepatocytes was not significantly different between recurrent and non-recurrent cases, both in tumoral and in non-tumoral tissues. Instead, the endothelial expression was significantly higher in the tumor vasculature of recurrent patients in comparison with non-recurrent patients (**Figure 1** and **Table 3**). The same expression pattern was also present in the validation cohort (**Table 3**).

**Table 3:** Intensity of angiopoietin-2 staining (expressed as optical density [OD]) in primary tumors of recurrent and non-recurrent cases of the training and validation cohorts, and in recurrent tumors occurring in transplanted livers, and metastasis of both training and validation cohorts.

Modena’s training cohort (*n* = 140)				Bologna’s validation cohort (*n* = 60)		
Recurrent group (n=68)		Non-recurrent group (n=68)	*p*-value	Recurrent group (n=30)	Non-recurrent group (n=30)	*p*-value
**Primary tumor**						
Endothelial (T)	0.435 ± 0.05	0.350 ± 0.06	<0.001	0.445 ± 0.01	0.400 ± 0.01	0.001
Hepatocyte (T)	0.308 ± 0.07	0.306 ± 0.09	0.768	0.398 ± 0.01	0.355 ± 0.04	0.098
Hepatocyte (NT)	0.410 ± 0.05	0.409 ± 0.05	0.612	0.441 ± 0.02	0.450 ± 0.02	0.432
**Recurrent hepatic tumor (*n* = 12)**						
Endothelial (T)	0.528 ± 0.060	–		–	–	
Hepatocyte (T)	0.552 ± 0.143	–		–	–	
Hepatocyte (NT)	0.560 ± 0.097	–		–	–	
**Extrahepatic metastasis (*n* = 9)**						
Lung (*n* = 4)						
Endothelial	0.645 ± 0.11	–		0.579 ± 0.02	–	
Parenchymal	0.652 ± 0.14	–		0.572 ± 0.03	–	
Bone (*n* = 2)						
Endothelial	0.576 ± 0.056	–				
Parenchymal	0.606 ± 0.042	–				
Lymph node (*n* = 3)						
Endothelial	0.790 ± 0.005	–				
Parenchymal	0.749 ± 0.006	–				

T, Tumor.

NT, Non-tumor.

The immunohistochemical analysis of recurrent hepatic HCC after LT and of extra-hepatic metastasis (nine patients) showed extremely high levels of angiopoietin-2 expression at endothelial level, higher than the primary tumor both for hepatic recurrence and for all the different types of metastasis; extra-hepatic metastasis also displayed very high angiopoietin-2 expression in metastatic hepatocytes (**Figure 1** and **Table 3**).

### Analysis for recurrence

HCC recurrence occurred in 31/70 (22.1%) patients within the first year from LT; 16/70 (11.4%) recurred in the second year from LT, and 23/70 (32.8%) recurred 3–5 years after LT.

Kaplan–Meier analysis showed that all the factors tested, except for gender and previous downstaging treatments, were significantly associated with the probability of HCC recurrence after LT (**Supplementary Figure 1**). Among the different factors examined, a higher relationship with the risk of recurrence was found for factors that were, either directly (such as endothelial angiopoietin-2 or microvascular invasion) or indirectly (such as AFP levels), related to biological HCC aggressiveness. The Milan criteria (*p* = 0.011) were significantly related to the probability of recurrence while the Metroticket score was not (*p* = 0.653). However, AFP inclusion in the Metroticket score (Metroticket_AFP) significantly improved its performance (*p* = 0.001). The AFP model also showed a good prediction of recurrence (*p* < 0.001).

**Figure 2:**
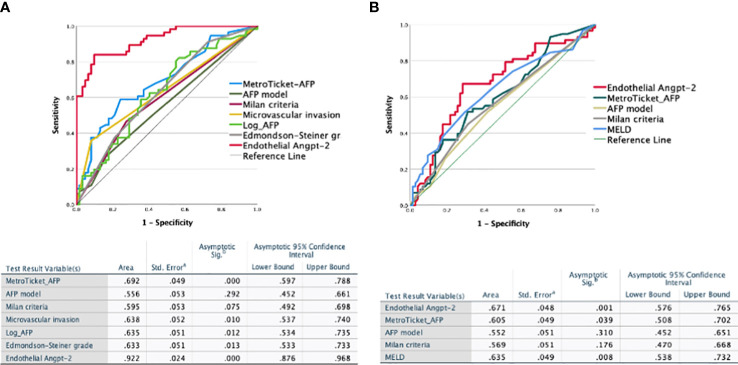
Receiver operating characteristic (ROC) analysis. Receiver operating characteristic (ROC) curves for the endothelial angiopoietin-2 and the different clinical scores for HCC recurrence **(A)** and survival **(B)** after liver transplantation in 140 matched transplanted HCC patients. angiopoietin-2 discriminating capacity was significantly higher than that of all clinical or pathologic scores for both recurrence **(A)** and survival **(B)**.

We evaluated the diagnostic power of the significant factors (Milan criteria, Metroticket_AFP, AFP model, presence of microvascular invasion, Edmondson–Steiner grade, log AFP value at the time of LT, and endothelial angiopoietin-2 expression in the explanted liver) by receiver operating characteristic (ROC) curve analysis to predict HCC recurrence after LT. Microvascular invasion, Metroticket_AFP, AFP model, and endothelial angiopoietin-2 significantly predicted recurrence. However, endothelial angiopoietin-2 expression had a much higher specificity and sensitivity for recurrence (AUC 0.922, 95% CI 0.876–0.962, *p* < 0.0001) than the other significant variables (**Figure 2A**). Similar figures were obtained for the validation cohort (AUC 0.957, 95% CI 0.862–1.000, *p* = 0.003).

The univariate Cox analysis identified the BMI, log AFP at transplant, endothelial angiopoietin-2 expression, Milan criteria, Metroticket_AFP score, and the AFP model as significantly associated with recurrence. In multivariate analysis, angiopoietin-2 expression was the only independent factor associated with recurrence (HR: 5.634, 95% CI 2.597–12.294, *p* < 0.001) (**Table 1**). These Cox analyses were confirmed in the validation cohort analysis (**Supplementary Table 2**).

### Survival analysis

At the time of the last visit, 77 patients (47.9%) had died. A significantly higher number of deaths occurred in recurrent vs. non-recurrent patients (58 [86.6%] vs. 9 [13.4%], respectively, *p* < 0.001). Survival was greatly influenced by recurrence, with recurrent patients having strikingly lower survival than non-recurrent patients (**Supplementary Figure 2**). Similar results were observed in the validation cohort, in which, among 19 deaths, 17 (89.4%) occurred in recurrent patients.

The number of deaths was higher in patients who relapsed in the first year vs. those recurring in the second or third to fifth year from LT (29/58 [50.0%] vs. 9/58 [15.5%] vs. 20/58 [34.3%], respectively). The median survival in these three groups was significantly different (17 vs. 51 vs. 75 months, respectively, *p* < 0.001, log-rank test) (**Supplementary Figure 2**).

The cause of death in the great majority of recurrent patients (86.8%) was due to the progression of the recurrent tumor, while the most frequent cause of death in non-recurrent patients was sepsis (66.7%).

No significant difference in mortality was observed among recurrent patients according to sex [male vs. female 40/46 (86.9%) vs. 2/2 (100.0%), *p* = 1.000, Fisher’s exact test].

The survival rate was borderline significant in patients with higher median AFP values at the time of the transplant (*p* = 0.070, log-rank test) while it was significantly lower in patients in the highest AFP quartile (i.e., 21 ng/ml) (*p* = 0.027, log-rank test). Median survival was also significantly lower in patients whose angiopoietin-2 expression in the endothelia of tumoral vasculature was higher than the median value (*p* < 0.001, log-rank test) as well as in patients with microvascular angioinvasion (*p* < 0.001, log-rank test) and in those with Edmondson G3–G4 (*p* = 0.007, log-rank test). The presence of portal vein thrombosis, previous therapeutic interventions for HCC, and sex or age at transplant were not significantly related to survival.

Survival rates were not significantly different between Milan-in and Milan-out patients (*p* = 0.138, Log-rank test) and patients stratified by the Metroticket (*p* = 0.775, log-rank test) or the AFP model. Incorporating the angiopoietin-2 value into the Milan criteria, i.e., coding as Milan-out those with upper median angiopoietin-2 levels, drastically improved survival prediction by Milan criteria (*p* = 0.003) (**Supplementary Figure 2**). Patients with a higher Metroticket_AFP score had only a moderate survival advantage (*p* = 0.022, log-rank test) (**Supplementary Figure 2**).

The UNIVARIATE Cox regression analysis showed that HCC recurrence, MELD, log-AFP at transplantation, Edmondson–Steiner grade, microvascular invasion, and endothelial angiopoietin-2 expressions were significantly associated with HCC recurrence after LT. According to the multivariate Cox analysis, Metroticket_AFP, MELD, and endothelial angiopoietin-2 expressions were independently related to survival (**Table 4**). AUROC also confirmed this for mortality: all factors tested in relation to survival, except for endothelial angiopoietin-2, Metroticket_AFP, and MELD, had a low discriminatory power (**Figure 2B**). Endothelial angiopoietin-2 had the best predictive power.

**Table 4:** Univariate and multivariate analysis results for the survival of HCC after LT in the training cohort.

Variables	Univariate analysis		Multivariate analysis	
	HR (95% CI)	*p*	HR (95% CI)	*p*
Gender	0.618 (0.267–1.434)	0.263		
BMI	0.947 (0.870–1.031)	0.211		
MELD	1.039 (1.000–1.079)	0.048	1.044 (1.004–1.086)	0.032
Log AFP at transplant *	1.574 (1.188–2.086)	0.002		
Endothelial angiopoietin-2 *,**, ***	2.622 (1.564–4.396)	0.000	2.274 (1.232–4.999)	0.009
Edmondson–Steiner grade**	1.651 (1.163–2.343)	0.005		
Microvascular invasion ***	2.987 (1.775–5.026)	0.000		
Milan score	0.698 (0.424–1.118)	0.132		
Metroticket_AFP Score	0.519 (0.299–0.901)	0.020	0.533 (0.302–0.942)	0.030
AFP model^	1.440 (0.654–3.174)	0.365		
Downstaging treatments before LT	1.062 (0.768–1.467)	0.717		

*, **, *** collinear.

^ ref. Duvoux et al. (10).

## Discussion

In this derivation–validation study of patients who underwent LT for HCC, we have shown, both in the training and in the validation cohorts, that endothelial expression of angiopoietin-2 assessed at explant was able to strongly and independently predict HCC recurrence and mortality after LT. Analysis of angiopoietin-2 in explanted livers strikingly improved the accuracy of outcome prediction in comparison with the available scores previously designed for this purpose, as shown both by Cox regression analysis and by AUROCs. Angiopoietin-2 staining was abundant both in hepatocyte cytoplasms and in the sinusoidal endothelia, but it was the latter that was highly significant in recurrent HCCs, highlighting the important role of endothelium in neoangiogenic activation. It should be emphasized that although these data were obtained on liver explant, they could be easily collected well before LT by ultrasound-guided liver biopsy at first HCC diagnosis, thus largely anticipating the characterization of the biologic aggressiveness of the individual HCC (15). The relevant gain in the prediction of HCC aggressiveness with its prognostic information can offset the risk associated with the biopsy maneuver. It should be underlined that severe complications, mostly hemorrhagic, have become rare, less than 0.5% with null mortality (18, 19). Also, the issue of sampling error is more likely to affect the histologic interpretation rather than the molecular one. The increasing sensitivity of the methodologies, such as Next-Generation Sequencing, is able to overcome the challenge of obtaining a representative tumor sample, bringing the analysis to the single-cell level (20). From the clinical point of view, our model for the prediction of HCC recurrence after LT based on angiopoietin-2 would be a useful tool to identify the best candidates for LT, optimize the radiological schedule of follow-up, and finally determine the optimal target population that could receive maximal benefit from novel adjuvant treatments blocking the angiopoietin-2 pathway.

The prediction of HCC recurrence after LT continues to represent an unsolved medical need. This has led, in recent years, to an intense search for clinical scores that could help in choosing the best candidates for LT and in the risk stratification of HCC recurrence and mortality after LT according to biochemical, radiological, and pathological features collected before and after LT. In the direct comparison for recurrence between endothelial angiopoietin-2 and clinical scores, endothelial angiopoietin-2, with an AUC of 0.922, greatly outperformed all clinical scores. Among the other scores, Metroticket_AFP showed the best performance. However, the AUC for the other scores were below 0.640. The predictive strength for recurrence of endothelial angiopoietin-2 was further confirmed by the Cox multivariate regression analysis, which showed that endothelial angiopoietin-2 was the only independent factor for post-LT HCC recurrence. The ability of angiopoietin-2 to predict recurrence is not surprising. We have already shown that angiopoietin-2 is the leader gene in the transcriptomic signature defining aggressiveness in HCC (14) and that this is linked to the powerful neoangiogenesis that develops in these patients. This is not unique to HCC. Similar data have been reported in both lung (21) and breast cancer (22).

Among the available prediction rules for survival, MetroTicket and MetroTicket_AFP were explicitly built to calculate the 5-year HCC survival after LT. Similarly to our model, the MetroTicket calculator is based on explant pathology, also including microvascular invasion, while MetroTicket_AFP is calculated according to the preoperative radiological staging and AFP levels. Kaplan–Meier analysis for survival indicated that of all clinical scores considered (Milan criteria, Metroticket_AFP, and AFP model) only Metroticket_AFP predicted survival These data point, with greater or lesser intensity depending on the factor considered, toward a greater relevance of the biologic rather than the morphologic or pathologic features for predicting recurrence or defining prognosis. The number and size of the tumor, like in the Milan criteria or Metroticket, are clearly insufficient for prognostication and only the addition of angiopoietin-2 for Milan criteria, or AFP for Metroticket, makes them able to discriminate between low and high risk of the event. Endothelial angiopoietin-2 strongly related to survival at the Kaplan–Meier analysis, as robustly as recurrence, which was understandably the most decisive post-transplant factor associated with survival. The strong relationship between endothelial angiopoietin-2 and survival was confirmed by the Cox regression analysis. In the multivariable model, Metroticket_AFP, MELD, and endothelial angiopoietin-2 were the only factors independently predicting survival after LT, but Angiopoietin had a higher level of significance. Compared with the other prediction rules, our model showed better discrimination and calibration for predicting survival after LT. The ROC curve analysis showed that endothelial angiopoietin-2 had higher accuracy in identifying non-survivors than all clinical scores tested, including MELD. These data indicate that a specific biologic feature of the tumor, i.e., endothelial angiopoietin-2, is more robust than any other clinical or pathologic factor tested for predicting survival after liver transplantation.

Angiopoietin-2 is a protein expressed in the endothelium and is involved in the processes of neoangiogenesis. Although it is a mainly endothelial gene, it can also have a secondary parenchymal localization (23, 24). Angiopoietin-2 is inducible by hypoxia and its expression is increased in the endothelial cells in this condition (15, 23, 25). Also, it is fundamental for the progression of HCC (26). This gene has been identified as the molecular signature leader gene of the five liver genes, strongly related to growth rate, neoangiogenesis, aggressiveness, and high metastatic capacity of HCC patients (14). When fresh tissue is not available for analysis, the histochemical demonstration of angiopoietin-2 in the liver is as accurate as the transcriptomic signature in characterizing aggressive HCCs (15) It is, therefore, not surprising that angiopoietin-2-positive HCCs had such a high rate of recurrence after liver transplant. On this same line, it is also not surprising that a high percentage of patients did not experience a recurrence in the liver but in an extra-hepatic site and a few years after. This opens the possibility of dormant micrometastases (27) that, for several different reasons, like modifications of immunocompetence or concurrent therapies, find a favorable environment for their growth in an organ different from the liver, and sometime after LT. Not surprisingly, although angiopoietin-2 was found to be the most powerful predictor of recurrence, many other factors, all expressions of greater tumor aggressiveness (such as AFP levels, size of the larger nodule, presence of microvascular invasion, poorly differentiated/undifferentiated grading), were significant on univariate analysis. In fact, relapsed HCCs had higher AFP levels, more undifferentiated grading, and higher tumor size than non-recurrent patients. The AFP level is known to be elevated in fast-growing, more aggressive HCCs than in slow-growing ones (14). An elevated AFP level has also been linked to the risk of relapse after liver transplantation (28).

In conclusion, our study demonstrated that endothelial angiopoietin-2 expression is the only independent predictor of post-liver transplant tumor recurrence, compared to other relevant characteristics of tumor aggressiveness such as tumor size HCC and/or presence of residual viability of the tumor nodule at explant. Equally important was the demonstration that angiopoietin-2 is an independent factor of mortality, emphasizing the extremely important role of the biology of the tumor in defining patients’ prognosis after liver transplantation. The availability of selective angiopoietin-2 inhibitors can open the path for clinical trials attempting to modify these patients’ otherwise extremely poor prognosis.

## Data availability statement

The raw data supporting the conclusions of this article will be made available by the authors, without undue reservation.

## Ethics statement

The studies involving human participants were reviewed and approved by AVEN Ethical Committee. The participants/participants provided their written informed consent to participate in this study, and was gathered in line with national legislation guidelines.

## Author contributions

SL, MR, AR, FM, RMC, FD, and SM: Performing the immunohistochemistry study, analysis of the results, review of the manuscript; FL, FR, SG, MS, AP, LDM, DR, NDM, BC, FS, and PM: creation of patients’ database, analysis of clinical data; AD’E, MC, FDB, AC, GG, and MM-C: analysis of data, critically reviewing the manuscript; CCe, ME, and CCa: statistical analysis; EV: design of the study; analysis of results, writing up the paper. All authors contributed to the article and approved the submitted version.

## Funding

This study was supported by the AIRC under IG 2020 ID. 24858 Project – P.I. EV.

## Conflict of interest

The authors declare that the research was conducted in the absence of any commercial or financial relationships that could be construed as a potential conflict of interest.

## Publisher’s note

All claims expressed in this article are solely those of the authors and do not necessarily represent those of their affiliated organizations, or those of the publisher, the editors and the reviewers. Any product that may be evaluated in this article, or claim that may be made by its manufacturer, is not guaranteed or endorsed by the publisher.

## References

[1] Dasgupta P Henshaw C Youlden DR Clark PJ Aitken JF Baade PD. Global trends in incidence rates of primary adult liver cancers: A systematic review and meta-analysis. Front Oncol (2020) 10:171. doi: 10.3389/fonc.2020.00171 32185125 PMC7058661

[2] White DL Thrift AP Kanwal F Davila J El-Serag HB. Incidence of hepatocellular carcinoma in all 50 united states, from 2000 through 2012. Gastroenterology (2017) 152:812–20.e5. doi: 10.1053/j.gastro.2016.11.020 27889576 PMC5346030

[3] Ripoll C Groszmann RJ Garcia-Tsao G Bosch J Grace N Burroughs A. Hepatic venous pressure gradient predicts development of hepatocellular carcinoma independently of severity of cirrhosis. J Hepatol (2009) 50:923–8. doi: 10.1016/j.jhep.2009.01.014 PMC3721146 19303163

[4] Masuzaki R Tateishi R Yoshida H Sato S Kato N Kanai F. Risk assessment of hepatocellular carcinoma in chronic hepatitis c patients by transient elastography. J Clin Gastroenterol (2008) 42:839–43. doi: 10.1097/mcg.0b013e318050074f 18668703

[5] Mazzaferro V Regalia E Doci R Andreola S Pulvirenti A Bozzetti F. Liver transplantation for the treatment of small hepatocellular carcinomas in patients with cirrhosis. N Engl J Med (1996) 334:693–9. doi: 10.1056/NEJM199603143341104 8594428

[6] Llovet JM Brú C Bruix J. Prognosis of hepatocellular carcinoma: The BCLC staging classification. Semin Liver Dis (1999) 19(3):329–38. doi: 10.1055/s-2007-1007122 10518312

[7] Kim WR Lake JR Smith JM Skeans MA Schladt DP Edwards EB. OPTN/SRTR 2015 annual data report: Liver. Am J Transplant (2017) 17:174–251. doi: 10.1111/ajt.14126 28052604

[8] Yao FY Ferrell L Bass NM Watson JJ Bacchetti P Venook A. Liver transplantation for hepatocellular carcinoma: Expansion of the tumor size limits does not adversely impact survival. Hepatology (2001) 33:1394–403. doi: 10.1053/jhep.2001.24563 11391528

[9] Mazzaferro V Llovet JM Miceli R Bhoori S Schiavo M Mariani L. Predicting survival after liver transplantation in patients with hepatocellular carcinoma beyond the Milan criteria: A retrospective, exploratory analysis. Lancet Oncol (2009) 10:35–43. doi: 10.1016/S1470-2045(08)70284-5 19058754

[10] Duvoux C Roudot-Thoraval F Decaens T Pessione F Badran H Piardi T. Liver transplantation for hepatocellular carcinoma: A model including alpha-fetoprotein improves the performance of Milan criteria. Gastroenterology (2012) 143:985–6. doi: 10.1053/j.gastro.2012.05.052 22750200

[11] Mazzaferro V Sposito C Zhou J Pinna AD De Carlis L Fan J. Metroticket 2.0 model for analysis of competing risks of death after liver transplantation for hepatocellular carcinoma. Gastroenterology (2018) 154(1):128–39. doi: 10.1053/j.gastro.2017.09.025 28989060

[12] de'Angelis N Landi F Carra MC Azoulay D. Managements of recurrent hepatocellular carcinoma after liver transplantation: A systematic review. World J Gastroenterol (2015) 21:11185–98. doi: 10.3748/wjg.v21.i39.11185 PMC4607916 26494973

[13] Parfitt JR Marotta P Alghamdi M Wall W Khakhar A Suskin NG. Recurrent hepatocellular carcinoma after transplantation: Use of a pathological score on explanted livers to predict recurrence. Liver Transpl (2007) 13:543–51. doi: 10.1002/lt.21078 17394152

[14] Villa E Critelli R Lei B Marzocchi G Cammà C Giannelli G. Neoangiogenesis-related genes are hallmarks of fast-growing hepatocellular carcinomas and worst survival. results from a prospective study. Gut (2016) 65:861–9. doi: 10.1136/gutjnl-2014-308483 25666192

[15] Faillaci F Marzi L Critelli R Milosa F Schepis F Turola E. Liver angiopoietin-2 is a key predictor of *De novo* or recurrent hepatocellular cancer after hepatitis c virus direct-acting antivirals. Hepatology (2018) 68:1010–24. doi: 10.1002/hep.29911 PMC6175123 29604220

[16] Edmondson HA Steiner PE. Primary carcinoma of the liver: A study of 100 cases among 48,900 necropsies. Cancer (1954) 7:462–503. doi: 10.1002/1097-0142(195405)7:3<462::aid-cncr2820070308>3.0.co;2-e 13160935

[17] Martins-Filho SN Paiva C Azevedo RS Alves VAF. Histological grading of hepatocellular carcinoma-a systematic review of literature. Front Med (Lausanne) (2017) 4:193. doi: 10.3389/fmed.2017.00193 29209611 PMC5701623

[18] Mulazzani L Terzi E Casadei G Pasquali V Felicani C Stefanini F. Retrospective analysis of safety of ultrasound-guided percutaneous liver biopsy in the 21st century. Eur J Gastroenterol Hepatol (2021) 33 1S Suppl 1:e355–62. doi: 10.1097/MEG.0000000000002080 35048647

[19] Jing H Yi Z He E Xu R Shi X Li L. Evaluation of risk factors for bleeding after ultrasound-guided liver biopsy. Int J Gen Med (2021) 14:5563–71. doi: 10.2147/IJGM.S328205 PMC8444981 34539186

[20] Martin SP Wang XW. The evolving landscape of precision medicine in primary liver cancer. Hepat Oncol (2019) 6(2):HEP12. doi: 10.2217/hep-2019-0004 31205678 PMC6566133

[21] Tsakogiannis D Nikolakopoulou A Zagouri F Stratakos G Syrigos K Zografos E. Update overview of the role of angiopoietins in lung cancer. Medicina (Kaunas) (2021) 57(11):1191. doi: 10.3390/medicina57111191 34833409 PMC8625006

[22] Ramanathan R Olex AL Dozmorov M Bear HD Fernandez LJ Takabe K. Angiopoietin pathway gene expression associated with poor breast cancersurvival. Breast Cancer Res Treat (2017) 162(1):191–8. doi: 10.1007/s10549-017-4102-2 PMC5290093 28062977

[23] Pichiule P Chavez JC LaManna JC. Hypoxic regulation of angiopoietin-2 expression in endothelial cells. J Biol Chem (2004) 279:12171–80. doi: 10.1074/jbc.M305146200 14702352

[24] Critelli R Milosa F Faillaci F Condello R Turola E Marzi L. Microenvironment inflammatory infiltrate drives growth speed and outcome of hepatocellular carcinoma: A prospective clinical study. Cell Death Dis (2017) 8:e3017. doi: 10.1038/cddis.2017.395 28837142 PMC5596578

[25] Goettsch W Gryczka C Korff T Ernst E Goettsch C Seebach J. Flow-dependent regulation of angiopoietin-2. J Cell Physiol (2008) 214:491–503. doi: 10.1002/jcp.21229 17960565

[26] Sugimachi K Tanaka S Taguchi K Aishima S Shimada M Tsuneyoshi M. Angiopoietin switching regulates angiogenesis and progression of human hepatocellular carcinoma. J Clin Pathol (2003) 56:854–60. doi: 10.1136/jcp.56.11.854 PMC1770094 14600132

[27] Sosa MS Bragado P Aguirre-Ghiso JA. Mechanisms of disseminated cancer celldormancy: An awakening field. Nat Rev Cancer (2014) 14(9):611–22. doi: 10.1038/nrc3793 PMC4230700 25118602

[28] Nörthen A Asendorf T Walson PD Oellerich M. Diagnostic value of alpha-1-Fetoprotein (AFP) as a biomarker for hepatocellular carcinoma recurrence after liver transplantation. Clin Biochem (2018) 52:20–5. doi: 10.1016/j.clinbiochem.2017.10.011 29054441

