# Supplementary material for: Endothelial angiopoietin-2 overexpression in explanted livers identifies subjects at higher risk of recurrence of hepatocellular carcinoma after liver transplantation
Source: Front Oncol. 2022 Sep 8;12:960808. doi: 10.3389/fonc.2022.960808 (PMC9493368; doi:10.3389/fonc.2022.960808)
Supplement: Supplementary file 1 [file DataSheet_1.pdf]

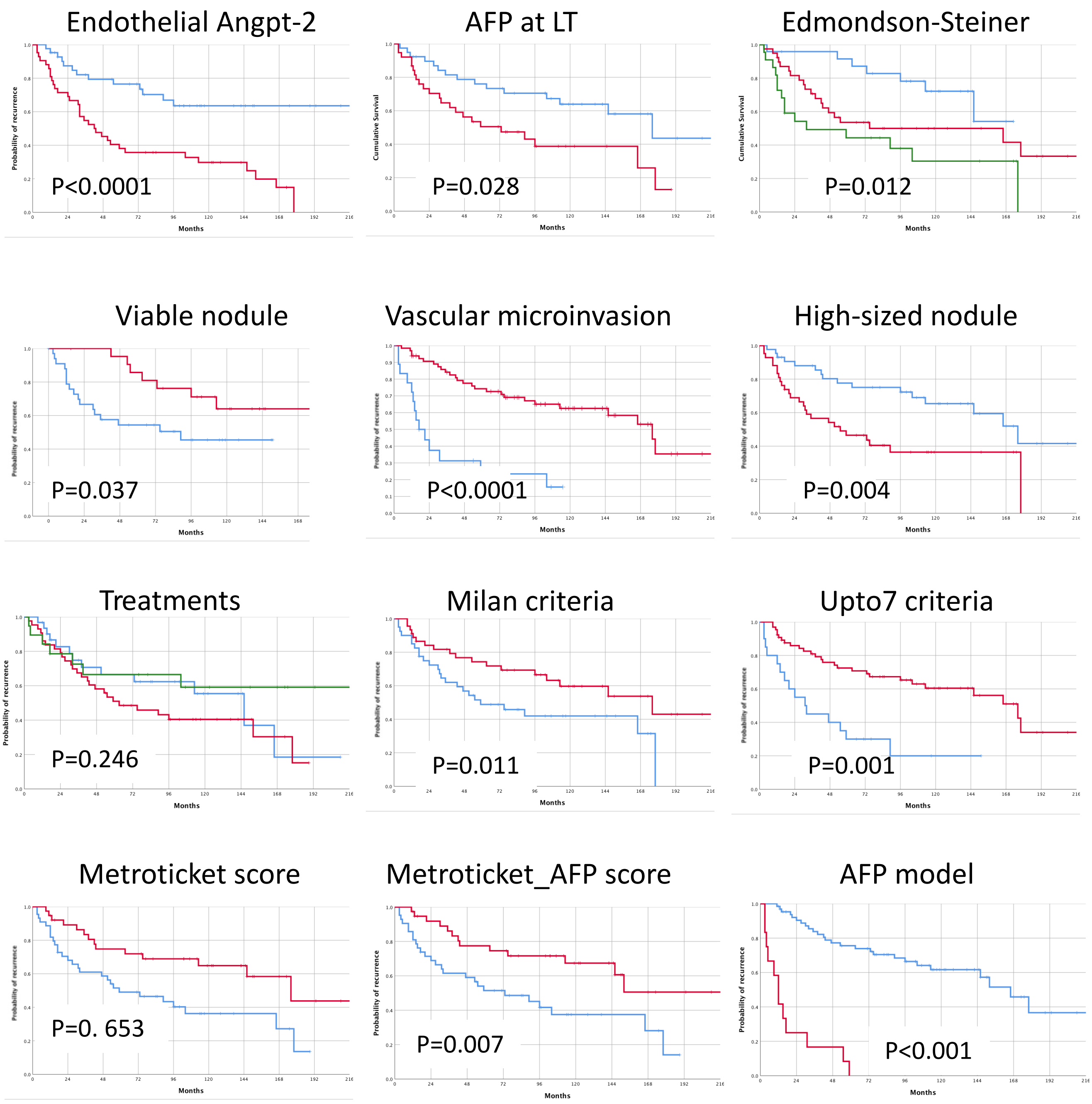

Probability of HCC recurrence after LT. Probability of HCC recurrence after liver transplantation was evaluated by Kaplan-Meier method. A. A clear-cut distinction was present between patients, who had higher median angiopoietin-2 levels, and those who had lower median levels. B-D. Subjects who at transplant had microvascular invasion in explanted liver or Edmondson-Steiner grade G3-G4 or higher AFP levels had high recurrence rate but absence of microvascular invasion, Edmondson-Steiner grade G0-G2 or lower AFP levels did not relate with recurrence -free outcome. E-H. Predictive power for the most used clinical scores was low. Among the scores, AFP model was the one that better stratified recurrence risk.
