# Supplementary material for: Endothelial angiopoietin-2 overexpression in explanted livers identifies subjects at higher risk of recurrence of hepatocellular carcinoma after liver transplantation
Source: Front Oncol. 2022 Sep 8;12:960808. doi: 10.3389/fonc.2022.960808 (PMC9493368; doi:10.3389/fonc.2022.960808)
Supplement: Supplementary file 2 [file DataSheet_2.pdf]

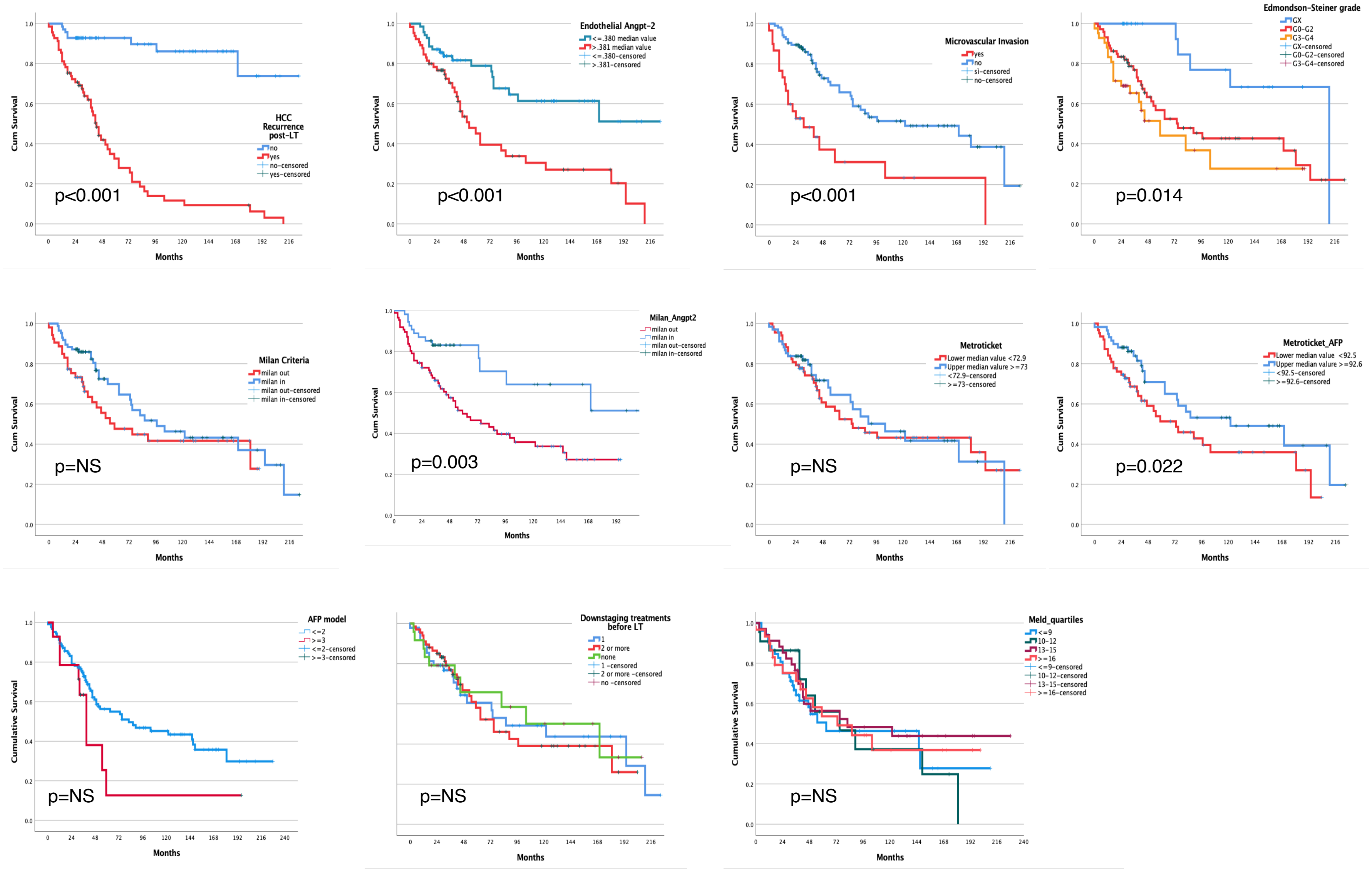

Probability of HCC survival after LT. Probability of HCC survivall after liver transplantation was evaluated by Kaplan-Meier method
