# Supplementary material for: Endothelial angiopoietin-2 overexpression in explanted livers identifies subjects at higher risk of recurrence of hepatocellular carcinoma after liver transplantation
Source: Front Oncol. 2022 Sep 8;12:960808. doi: 10.3389/fonc.2022.960808 (PMC9493368; doi:10.3389/fonc.2022.960808)
Supplement: Supplementary file 3 [file Table_1.docx]

Supplementary Table 1 - Univariate and multivariate analysis results for recurrence of HCC after LT in the validation cohort

|  | **Univariate analysis** |  | **Multivariate analysis** |  |
| --- | --- | --- | --- | --- |
| **Variables** | **HR (95% CI)** | **p** | **HR (95% CI)** | **p** |
| **Model 1** |  |  |  |  |
| Gender | 0.736 (0.235-2.142) | 0.574 |  |  |
| BMI | 0.937 (0.825-1.064) | 0.318 |  |  |
| MELD score | 0.950 (0.886-1.018) | 0.143 |  |  |
| Log AFP LT | 1.195 (0.634-2.255) | 0.582 |  |  |
| Endothelial Angiopoietin-2*,**,^ | **5.678 (1.501-21.475)** | **0.011** | **6.444 (1.340-30.974)** | **0.020** |
| Edmondson-Steiner grade* | 1.698 (0.932-3.093) | 0.084 |  |  |
| Microvascular invasion** | **4.814 (1.623-14.275)** | **0.005** |  |  |
| Milan Criteria | **0.436 (0.193-0.988)** | **0.047** | 0.594 (0.046-7.614) | 0.689 |
| Metroticket_AFP Score | 0.981 (0.957-1.006) | 0.140 |  |  |
| AFP model^ | 2.135 (0.919-4.961) | 0.078 | 3.411 (0.552-21.070) | 0.187 |
| Number of downstaging treatments before LT | **2.581 (1.166-5.713)** | **0.019** | 2.379 (0.362-15.647) | 0.367 |
| **Model 2** |  |  |  |  |
| Edmondson-Steiner grade | 1.698 (0.932-3.093) | 0.084 | 1.603 (0.447-5.742) | 0.469 |
| Microvascular invasion | **4.814 (1.623-14.275)** | **0.005** | **5.799 (1.411-23.832)** | **0.015** |
| Milan Criteria | **0.436 (0.193-0.988)** | **0.047** | 1.508 (0.487-4.670) | 0.477 |
| AFP model | 2.135 (0.919-4.961) | 0.078 | 0.997 (0.320-3.107) | 0.996 |

*, **, ° , ^ collinear
