# Supplementary material for: Endothelial angiopoietin-2 overexpression in explanted livers identifies subjects at higher risk of recurrence of hepatocellular carcinoma after liver transplantation
Source: Front Oncol. 2022 Sep 8;12:960808. doi: 10.3389/fonc.2022.960808 (PMC9493368; doi:10.3389/fonc.2022.960808)
Supplement: Supplementary file 4 [file Table_2.docx]

Supplementary Table 2 - Univariate and multivariate analysis results for survival of HCC after LT in the validation cohort

|  | **Univariate analysis** |  | **Multivariate analysis** |  |
| --- | --- | --- | --- | --- |
| **Variables** | **HR (95% CI)** | **p** | **HR (95% CI)** | **p** |
| Gender | 1.617 (0.462-5.666) | 0.452 |  |  |
| BMI | 0.898 (0.858-1.150) | 0.886 |  |  |
| MELD | 0.998 (0.928-1.074) | 0.965 |  |  |
| Log AFP at transplant * | 0.762 (0.284-2.045) | 0.589 |  |  |
| **Endothelial Angiopoietin-2 *^,^ **^,^ ***** | **4.511 (1.185-17.182)** | **0.027** | **2.651 (1.642-4.277)** | **<0.001** |
| Edmondson-Steiner grade** | 1.539 (0.743-3.189) | 0.246 |  |  |
| Microvascular invasion*** | **12.614 (1.644-96.770)** | **0.015** |  |  |
| Milan score | **0.170 (0.047-0.612)** | **0.007** | 0.703 (0.446-1.109) | 0.130 |
| Metroticket _AFP Score | 0.400 (0.126-1.263) | 0.118 |  |  |
| AFP model^ | 1.757 (0.572-5.396) | 0.325 |  |  |
| Down staging treatments before LT | 2.144 (0.777-5.914) | 0.141 |  |  |

°, *, **, *** collinear

^ ref. Duvoux ^10^
